# Integrative Transcriptomic, Proteomic and Epigenetic Analysis Uncovers Reproductive Dysregulation in F1 Males of *Solea senegalensis*

**DOI:** 10.3390/ijms27052153

**Published:** 2026-02-25

**Authors:** Marco Anaya-Romero, Alberto Arias-Pérez, Daniel Ramírez, María Esther Rodríguez, Manuel Alejandro Merlo, Silvia Portela-Bens, Ismael Cross, Diego Robledo, Laureana Rebordinos

**Affiliations:** 1 Area of Genetics, Faculty of Marine and Environmental Sciences, Institute of Marine Research (INMAR), University of Cádiz, 11510 Cádiz, Spain; marco.anaya@unisimon.edu.co (M.A.-R.); alberto.arias@uca.es (A.A.-P.); daniel.ramirez@uca.es (D.R.); mariaesther.rodriguez@uca.es (M.E.R.); alejandro.merlo@uca.es (M.A.M.); silvia.portela@uca.es (S.P.-B.); ismael.cross@uca.es (I.C.); 2 Life Sciences Research Center, Faculty of Basic and Biomedical Sciences, Universidad Simón Bolívar, Barranquilla 080005, Colombia; 3 The Roslin Institute and Royal (Dick) School of Veterinary Studies, University of Edinburgh, Edinburgh EH25 9RG, UK; diego.robledo@roslin.ed.ac.uk

**Keywords:** *Solea senegalensis*, flatfish, transcriptomics, proteomics, DNA methylation, epigenetics, multi-omics integration, reproductive dysfunction

## Abstract

Reproductive dysfunction in captive-bred males of the flatfish *Solea senegalensis* remains a major bottleneck for its aquaculture. To clarify the molecular basis underlying these impairments, we performed an integrated analysis of transcriptomes, proteomes and methylomes from gonads of wild-type individuals and first-generation (F1) captive fish of both sexes. Nineteen RNA-seq libraries and eighteen LC–MS/MS proteomes were generated, allowing the quantification of more than 32,000 genes and 2221 proteins. Differential expression and principal component analyses revealed that sex was the primary driver of molecular variation, whereas origin (F1 vs. wild-type) had a more moderate effect. Multi-omics integration showed a partial and comparison-dependent correspondence between RNA and protein levels, with a marked RNA–protein decoupling in F1 males. Despite this limited concordance, functional enrichment analyses identified consistent regulation of key biological processes, including translation, energy metabolism, and reproductive pathways such as gametogenesis, fertilization, and early embryonic development. Within this regulatory framework, previously characterized DNA methylation landscapes in gonadal tissue suggest an additional epigenetic layer modulating the transcriptional potential of reproductive genes, particularly in captive-bred males. F1 males exhibited coordinated down-regulation of reproductive functions across omic layers, consistent with altered post-transcriptional and post-translational regulation. Overall, this study provides the first comprehensive multi-omics framework integrating transcriptomic, proteomic, and epigenetic information in S. senegalensis gonads, offering mechanistic insights into the molecular basis of reproductive dysfunction in F1 broodstock and supporting future strategies to improve reproductive performance in aquaculture.

## 1. Introduction

The Senegalese sole (*Solea senegalensis*) is a flatfish species of high commercial value and one of the most promising candidates for marine aquaculture diversification in southern Europe. Its strong market demand, high product value, and good adaptation to intensive and semi-intensive farming systems have driven sustained research efforts aimed at optimizing its production cycle [1]. Despite significant advances in larval rearing, nutrition, and grow-out performance, the closure of the full reproductive cycle under captive conditions remains a major bottleneck for the expansion and sustainability of its aquaculture.

One of the most persistent constraints in *S. senegalensis* farming is the poor reproductive performance of first-generation captive-bred males (F1), which severely limits spawning success. F1 males typically exhibit reduced sperm volume, altered sperm quality, and diminished fertilization capacity, resulting in low fertilization rates and compromised embryo viability. These deficiencies have been associated with functional alterations affecting late spermiogenic maturation and sperm competence, including processes involved in sperm motility, membrane remodeling, energy metabolism, and molecular programs associated with sperm–egg interaction, rather than severe structural abnormalities of the sperm flagellum [2]. Consequently, commercial hatcheries continue to rely largely on wild-caught males to ensure viable spawns, highlighting the incomplete domestication of the species.

Reproductive dysfunction in captive-bred fish is a widespread phenomenon in aquaculture and has been reported across multiple taxa, particularly in species with complex reproductive behaviors or strict environmental requirements [3]. In *S. senegalensis*, impaired reproduction is considered one of the main obstacles to achieving full domestication [4]. Captive-bred males frequently show alterations in testicular structure, reduced steroidogenic activity, differences in gonadal maturation, and molecular changes affecting spermatogenesis, sperm motility, and sperm integrity [5]. These alterations suggest that reproductive failure in F1 males is not merely a behavioral issue but rather reflects deeper physiological and molecular dysregulation within the gonads, potentially involving multiple layers of gene regulation.

At the molecular level, transcriptomic approaches have provided valuable insights into the mechanisms underlying reproductive dysfunction in *S. senegalensis*. RNA sequencing studies comparing wild-type and captive individuals, sexes, and reproductive stages have identified differential expression of genes involved in steroidogenesis, gametogenesis, cell cycle regulation, and stress response pathways, highlighting the influence of origin, sex, and maturity on gonadal gene expression [6,7]. In parallel, proteomic analyses of Senegalese sole testes have revealed changes in protein abundance between wild-type and F1 males, particularly during late maturation stages. These studies have implicated alterations in redox regulation, sperm motility machinery, and protein turnover systems, including the ubiquitin–proteasome pathway, as potential contributors to reduced fertility in captive males [8]. While these studies have identified key genes and proteins associated with reproductive impairment, they also highlight that transcriptional changes do not always translate into proportional protein-level responses, suggesting the involvement of post-transcriptional and post-translational regulatory mechanisms.

Although transcriptomic and proteomic approaches have been widely applied in reproductive biology, their combined analysis in *S. senegalensis* provides specific insight into how captivity-related conditions affect the concordance and decoupling between mRNA and protein expression in gonadal tissue. Integrative analyses were used to identify biological processes in which transcriptional changes are not fully translated into protein-level responses, particularly in F1 males, highlighting functional regulatory disruptions associated with reduced reproductive performance [9].

In this regulatory context, epigenetic mechanisms such as DNA methylation have emerged as important modulators of gene expression in fish reproduction. In *S. senegalensis*, DNA methylation analyses in gonadal tissues have shown that methylation patterns vary according to origin (wild-type versus captive-bred), sex, and sexual maturity stage, and are associated with genes involved in gonadal development and reproductive function [7,10]. These findings suggest that captivity-associated environmental conditions may induce epigenetic reprogramming that shapes the transcriptional potential of reproductive genes, particularly in males. However, how such epigenetic signatures relate to downstream protein expression and functional output remains largely unexplored.

In the present study, we integrate transcriptomic, proteomic and methylome analyses of gonadal tissue from wild-type and first-generation captive-bred *S. senegalensis*, considering both sexes, to characterize the molecular mechanisms underlying reproductive dysfunction in captivity. By combining differential expression, correlation analyses, and functional enrichment approaches, this work aims to elucidate the extent of concordance and decoupling between mRNA and protein levels, identify key biological processes affected by sex and origin, and provide a mechanistic framework to better understand the molecular basis of reduced reproductive efficiency in cultured Senegalese sole.

## 2. Results

### 2.1. General Transcriptomic and Proteomic Statistics

A total of 19 RNA-seq libraries were generated from gonadal tissues of first-generation captive-bred (F1) and wild-type (wt) *S. senegalensis* individuals. The total number of raw reads reached 1.04 × 10^9^, of which more than 98.7% remained as clean reads after quality filtering, resulting in 1.03 × 10^9^ reads retained for transcriptomic analysis. Overall sequencing quality was high, with a mean Q30 value of 90.7% and an average GC content of 48.8%, reflecting the high reliability of the sequencing data (Table S1). In parallel, LC–MS/MS-based proteomic analysis enabled the identification of a total of 2221 proteins across the 18 gonadal samples. After applying filtering criteria (≥2 unique peptides, removal of contaminants, and exclusion of proteins missing in more than 30% of biological replicates), 2046 proteins were consistently quantified in at least 70% of the samples. The number of quantified proteins per sample ranged from 1875 to 2104 (mean = 1978; standard deviation = 85), confirming the technical reproducibility of the proteomic analysis [11].

### 2.2. Principal Component Analysis (PCA) Schemes

**Figure 1:**
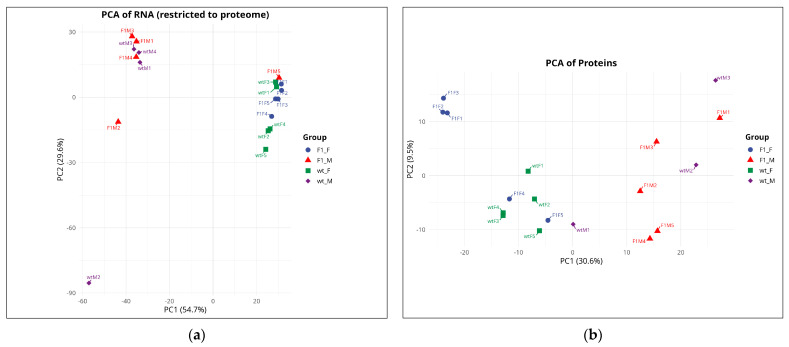
Principal component analysis (PCA) of the transcriptome (**a**) and proteome (**b**). Each point represents a biological replicate: F1F (blue circles), F1M (red triangles), wtF (green squares), and wtM (purple diamonds). F1F: first-generation captive-bred female, wtF: wild-type female, F1M: first-generation captive-bred male, and wtM: wild-type male.

Principal component analysis (PCA) of the transcriptomic dataset, restricted to genes with corresponding protein information, explained 84.3% of the total variance (PC1 = 54.7%, PC2 = 29.6%) and revealed a dominant segregation by sex (Figure 1a). Female samples (F1F and wtF) clustered predominantly toward positive PC1 values, whereas male samples (F1M and wtM) were located toward negative PC1 values, indicating a pronounced transcriptional divergence between sexes. Within this overall pattern, two notable exceptions were observed: sample F1M5 clustered within the female group (PC1 > 0), and wtM2 appeared as a distant outlier. The remaining biological replicates showed strong within-group consistency, forming compact clusters for F1F and wtF, whereas male samples (particularly wtM) displayed greater dispersion, indicative of higher biological heterogeneity.

PCA of the proteomic dataset explained 40.1% of the total variance (PC1 = 30.6%, PC2 = 9.5%) and similarly identified sex as the main axis of variation (Figure 1b). In this case, female samples were primarily located at negative PC1 values, whereas male samples clustered toward positive PC1 values, with wtM1 positioned close to the origin as an exception. The effect of origin (F1 vs. wild-type) was secondary and more evident in females, with F1F tending toward higher PC2 values compared to wtF. In males, origin-related patterns were less distinct due to greater dispersion, with wtM3 shifted toward the quadrant defined by higher PC1 and PC2 values. Overall, the concordance between transcriptomic and proteomic PCAs identifies sex as the primary source of molecular variation, with origin exerting a comparatively weaker effect, consistent with the greater number of DEGs and DEPs observed in sex-based comparisons relative to origin-based contrasts.

### 2.3. Identification of DEGs and DEPs

**Figure 2:**
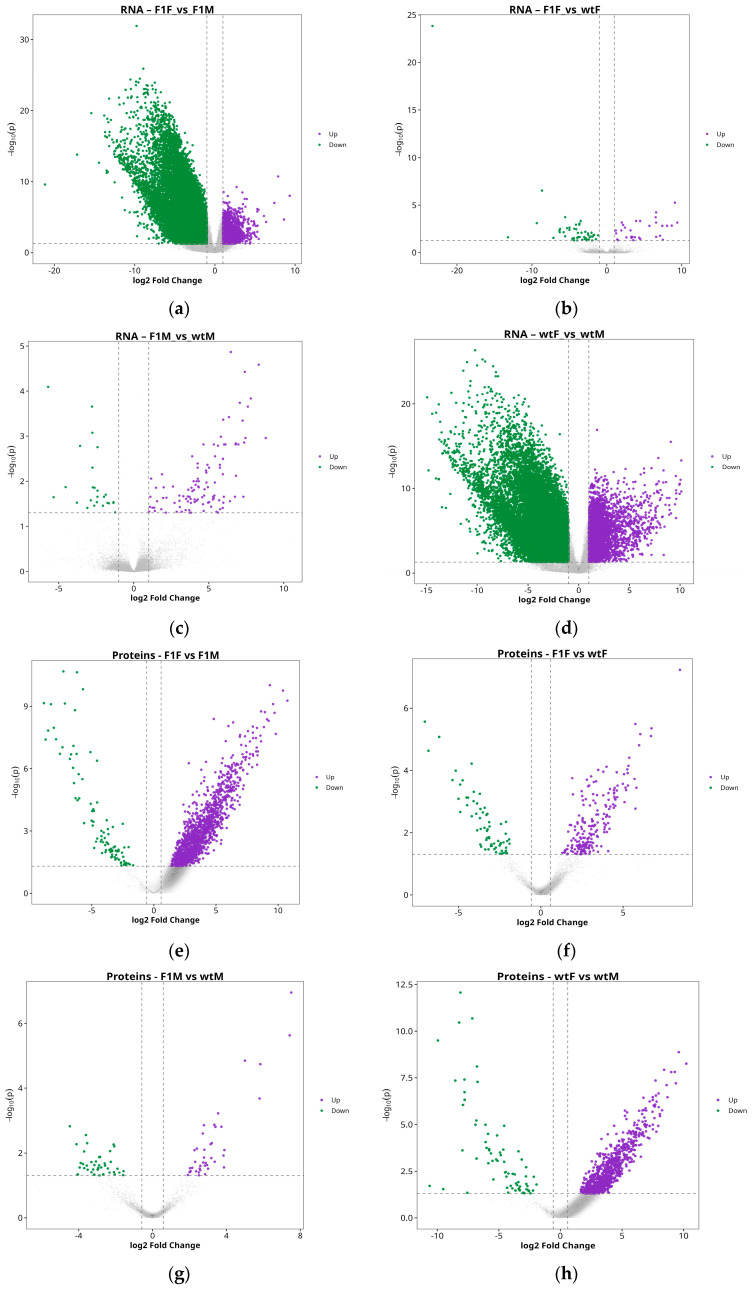
Differential expression analysis. Volcano plots of genes (**a**–**d**) and proteins (**e**–**h**) for the comparisons F1F vs. F1M, F1F vs. wtF, F1M vs. wtM, and wtF vs. wtM. Purple dots indicate upregulated entities, green dots indicate downregulated entities, and gray dots represent non-significant (NS) features. F1F: first-generation captive-bred female, wtF: wild-type female, F1M: first-generation captive-bred male, and wtM: wild-type male.

Differential expression analysis within the matched gene–protein set revealed a clear predominance of sex-related differences over those associated with origin. In the comparison between F1 females and males (F1F vs. F1M), a total of 971 differentially expressed genes (DEGs) were identified (308 upregulated and 663 downregulated) (Figure 2a), together with 1336 differentially expressed proteins (DEPs) (1243 upregulated and 93 downregulated) (Figure 2e), indicating pronounced transcriptomic and proteomic divergence between sexes in F1 individuals.

In contrast, the comparison between F1 and wild-type females (F1F vs. wtF) showed a much more limited number of changes, with 50 DEGs (14 upregulated and 36 downregulated) (Figure 2b) and 268 DEPs (202 upregulated and 66 downregulated) (Figure 2f).

Similarly, the comparison between F1 and wild-type males (F1M vs. wtM) revealed only 27 DEGs (13 upregulated and 14 downregulated) (Figure 2c) and 85 DEPs (41 upregulated and 44 downregulated) (Figure 2g), confirming a reduced impact of origin on male gonadal molecular profiles.

Finally, the comparison between wild-type females and males (wtF vs. wtM) yielded the highest number of differences, with 1253 DEGs (532 upregulated and 721 downregulated) (Figure 2d) and 913 DEPs (842 upregulated and 71 downregulated) (Figure 2h), further reinforcing sexual dimorphism as the primary source of molecular variation in the gonads of this species.

**Figure 3:**
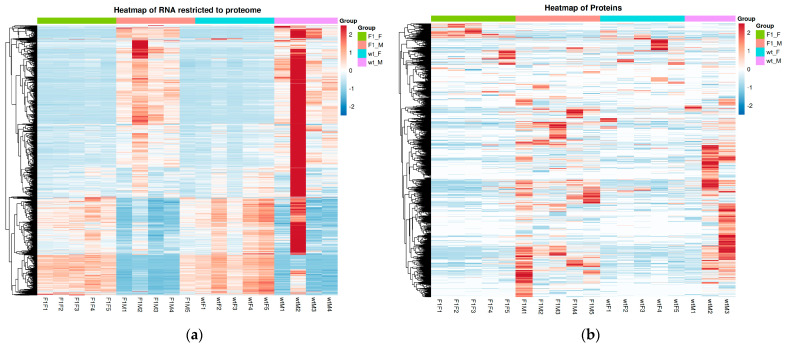
(**a**) Heatmap of genes restricted to the set matched with the proteomes, and (**b**) heatmap of proteins, highlighting a dominant segregation driven by sex and increased heterogeneity among male samples. F1F: first-generation captive-bred female, wtF: wild-type female, F1M: first-generation captive-bred male, and wtM: wild-type male. Rows correspond to genes or proteins, respectively, and columns represent individual samples. Expression levels are shown as standardized values (z-scores), with red and blue colors indicating higher and lower relative abundance, respectively. Hierarchical clustering was performed based on the similarity of expression profiles, and the top annotation bar indicates the experimental groups.

The pattern described above, was further supported by hierarchical clustering analyses represented in heatmaps, which confirmed sex as the main determinant of transcriptomic and proteomic variation in *S. senegalensis* gonads. Regarding RNA analysis (Figure 3a), a clear segregation was observed between females (F1F and wtF) and males (F1M and wtM), with largely consistent expression profiles within each group, except for a few outliers (e.g., wtM2 and F1M5) that displayed divergent patterns relative to their respective replicates. Most differentially expressed genes were organized into well-defined expression blocks, with a marked downregulation of multiple transcripts in male groups.

Similarly, the protein heatmap (Figure 3b) reproduced the sex-based separation, although with greater intragroup heterogeneity, particularly among wild-type males, indicating increased regulatory decoupling at the proteomic level. Nevertheless, at both omic layers, females exhibited more consistent expression profiles, whereas males showed greater dispersion, in agreement with the variability observed in PCA analyses. These results suggest that gonadal regulation in *S. senegalensis* males is subject to higher biological heterogeneity compared with females.

### 2.4. RNA–Protein Integration Analysis

**Figure 4:**
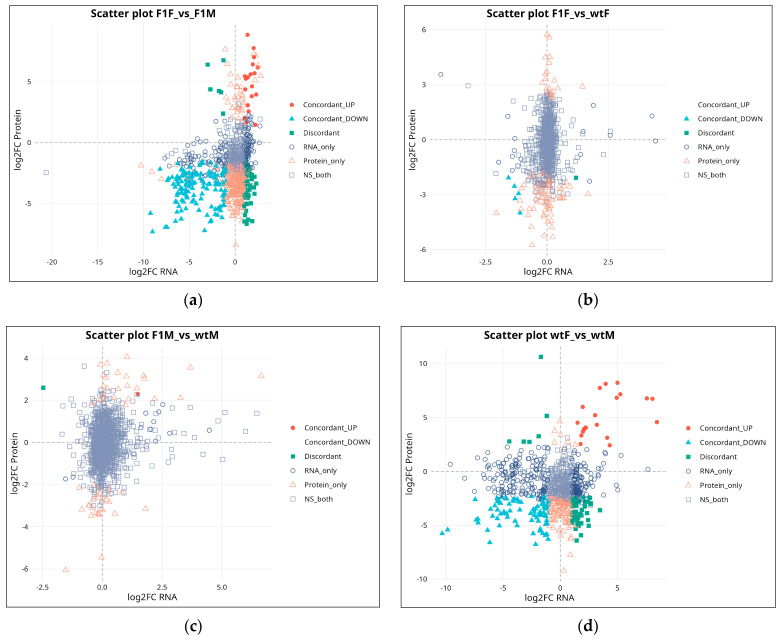
Correlation between transcriptomic and proteomic expression changes in *Solea senegalensis*. Scatter plots show the relationship between log_2_ fold-change (log_2_FC) values at the RNA and protein levels for the comparisons (**a**) F1F vs. F1M, (**b**) F1F vs. wtF, (**c**) F1M vs. wtM, and (**d**) wtF vs. wtM. Each point represents a mapped gene–protein pair and is classified according to its regulatory pattern: concordantly upregulated (red), concordantly downregulated (blue), discordant (green), RNA-exclusive (open circles), protein-exclusive (open triangles), or non-significant at both levels (gray squares). F1F: first-generation captive-bred female, wtF: wild-type female, F1M: first-generation captive-bred male, and wtM: wild-type male.

Integration of transcriptomic and proteomic datasets revealed that the correspondence between both omic layers was partial and varied depending on the comparison analyzed. In the F1F vs. F1M contrast (*n* = 867) (Figure 4a), the highest correlation was observed (r = 0.25; ρ = 0.24), with 161 concordant gene–protein pairs (18.6%), indicating a relatively coherent differential regulation between sexes in F1 individuals.

In the F1F vs. wtF comparison (*n* = 835), correlation values were low (r = 0.086; ρ = 0.139), and concordance was minimal, restricted to only five pairs (0.6%), with a predominance of signals exclusive to either RNA or protein levels (Figure 4b). In the F1M vs. wtM comparison (*n* = 1228), a modest correlation was detected (r = 0.187; ρ = 0.155), but concordance was virtually absent, with only one concordant pair (0.08%), reflecting a pronounced decoupling between transcriptome and proteome in F1 males relative to wild-type males (Figure 4c).

Finally, in the wtF vs. wtM comparison (*n* = 798), correlation was weak (r = 0.104; ρ = 0.00), and 102 concordant pairs (12.8%) were identified, together with a high number of exclusive or discordant entities (Figure 4d). This pattern characterizes sexual dimorphism under wild conditions as being strongly regulated by mechanisms that are largely non-concordant between RNA and protein levels.

### 2.5. Functional Analysis Using Gene Ontology

**Figure 5:**
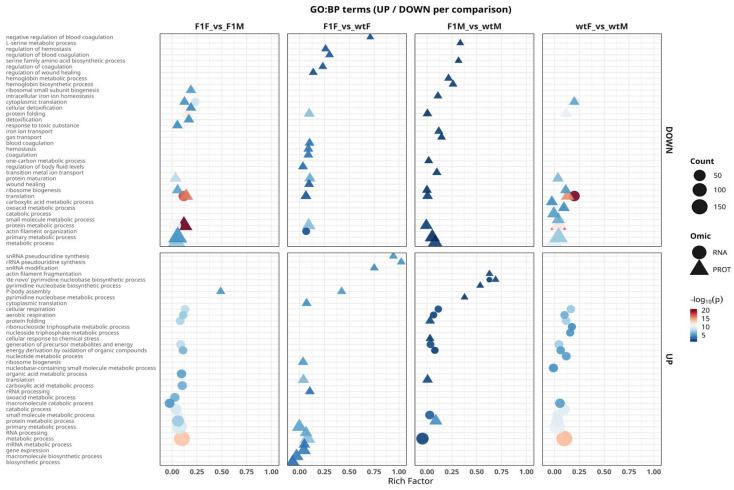
Integrated functional analysis of differentially expressed genes (RNA) and proteins (PROT) in *Solea senegalensis* gonads based on the Gene Ontology Biological Process (GO:BP) category. Significantly enriched terms that are positively regulated (UP, **lower panels**) and negatively regulated (DOWN, **upper panels**) in the comparisons F1F vs. F1M, F1F vs. wtF, F1M vs. wtM, and wtF vs. wtM are shown. Circular symbols represent terms derived from the transcriptome (RNA), whereas triangles correspond to the proteome (PROT). The x-axis indicates the rich factor (proportion of genes/proteins annotated to each term), symbol size reflects the number of associated entities (count), and the color scale represents the level of statistical significance (−log_10_ adjusted *p*-value). F1F: first-generation captive-bred female, wtF: wild-type female, F1M: first-generation captive-bred male, and wtM: wild-type male.

Integrated functional analysis of differentially expressed genes and proteins showed that molecular differences between sexes and origins in *S. senegalensis* are primarily driven by key biological processes (Figure 5). Across all comparisons, transcriptomic and proteomic profiles converged on a conserved functional signature dominated by pathways involved in protein synthesis, folding, and turnover, including translation, ribosome biogenesis, proteasome-mediated catabolic processes, and oxidoreductase activity. Together, these results highlight ribosomal and proteasomal pathways as central regulators of gonadal physiology, underlying both sexual dimorphism and origin-related molecular variation.

In the F1F vs. F1M comparison, a dichotomous functional pattern was observed, characterized by a generalized repression of translational processes (translation, ribosome biogenesis, protein maturation) concomitant with the activation of biosynthetic pathways (macromolecule biosynthetic process, gene expression). This profile reflects molecular sexual dimorphism in F1 individuals, with females displaying a more dynamic biosynthetic and metabolic state. In the F1F vs. wtF comparison, overlap between RNA and protein levels was limited. Both omic layers showed enrichment of metabolic functions (carboxylic acid metabolic process, energy derivation by oxidation of organic compounds); however, proteins were preferentially associated with chemical stress responses (cellular response to chemical stress, detoxification), whereas the transcriptome was mainly enriched in anabolic pathways related to nucleotide and cofactor biosynthesis. This contrast suggests that F1 females may compensate for increased energetic demands through enhanced oxidative defense mechanisms, likely associated with captive rearing conditions.

The F1M vs. wtM comparison displayed the most asymmetric functional pattern among all contrasts, with a clear disconnect between transcriptomic and proteomic signals. At the transcriptomic level, pathways related to RNA processing and degradation (RNA processing, rRNA processing, RNA catabolic process) predominated, whereas the proteome showed activity restricted to catabolic functions and small-molecule metabolism (protein metabolic process, energy derivation by oxidation of organic compounds). The absence of shared terms and the predominance of non-concordant signals confirm a marked functional decoupling between transcriptome and proteome in F1 males.

Finally, in the wtF vs. wtM comparison, the functional profile resembled that observed in F1F vs. F1M, with concordance between omic layers in ribosomal and proteasomal pathways (translation, protein folding, ribosome biogenesis), together with the additional enrichment, at the RNA level, of processes related to oxidative phosphorylation and aerobic respiration (oxidative phosphorylation, aerobic respiration), which are characteristic of physiological sexual dimorphism in flatfish species.

Overall, GO Biological Process analysis shows molecular regulation of *S. senegalensis* gonads organized around a shared biosynthetic and energetic control axis, whereas domestication effects manifest as a loss of synchrony between transcription and translation, particularly in F1 males, evidenced by the limited functional correspondence between RNA and protein levels. Functional analyses for the Cellular Component (CC) and Molecular Function (MF) categories are provided in Figure S1 and Figure S2, respectively.

#### 2.5.1. Functional Analysis of GO Terms Related to Reproductive Processes

**Figure 6:**
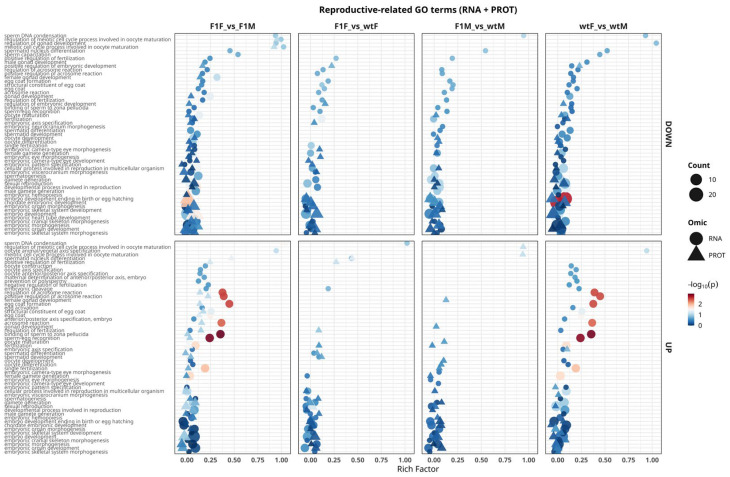
Functional analysis of Gene Ontology (GO) terms associated with reproductive processes in *Solea senegalensis* gonads integrating transcriptomic (RNA) and proteomic (PROT) data. GO Biological Process (GO:BP) terms related to gametogenesis, fertilization, gonadal development, and embryogenesis identified in the comparisons F1F vs. F1M, F1F vs. wtF, F1M vs. wtM, and wtF vs. wtM are shown. **Upper panels** represent downregulated (DOWN) terms, whereas **lower panels** show upregulated (UP) terms. Circles correspond to processes enriched at the RNA level, and triangles indicate those detected at the protein level. The x-axis represents the rich factor (proportion of genes/proteins annotated to each term relative to the total), symbol size reflects the number of entities involved (count), and the color scale indicates statistical significance (−log_10_ adjusted *p*-value). F1F: first-generation captive-bred female, wtF: wild-type female, F1M: first-generation captive-bred male, and wtM: wild-type male.

The reproductive-related GO terms panel (Figure 6) showed that the most consistent signals emerged in sex-based comparisons (F1F vs. F1M and wtF vs. wtM), where the highest density of terms associated with gametogenesis and fertilization was observed. Prominent processes included male gamete generation, female gamete generation, spermatogenesis, spermatid development/differentiation, oocyte maturation, and oocyte differentiation, together with events mediating sperm–oocyte interaction such as acrosome reaction, positive/negative regulation of acrosome reaction, sperm–egg recognition, binding of sperm to the zona pellucida, fertilization, and prevention of polyspermy.

Terms related to gonadal development (gonad development, female/male gonad development and their regulation) and early embryogenesis (embryo development, embryonic organ morphogenesis, embryonic axis specification, embryonic hemopoiesis) were also detected, indicating that sexual dimorphism affects not only gametogenic pathways but also covaries with developmental programs associated with reproductive success. At the omic level, most of these signals were predominantly detected at the transcriptomic level, with more limited proteomic support, mainly for terms closely related to gamete function (e.g., acrosome reaction, egg coat and structural constituent of egg coat).

In origin-based contrasts (F1F vs. wtF and, more markedly, F1M vs. wtM), reproductive-related terms were fewer and of lower magnitude, suggesting that origin (F1 vs. wild-type) more strongly modulates metabolic and structural processes than reproductive pathways per se. Nevertheless, in the F1M vs. wtM comparison, a persistent transcriptomic core related to fertilization (acrosomal function, sperm–oocyte recognition and binding) was detected, with limited concomitant translation at the protein level. This pattern is consistent with the transcriptome–proteome decoupling previously described for F1 males. Overall, these results identify gametogenesis, fertilization, and gonadal development pathways as the main functional axes underlying sex-based separation, whereas the effect of origin is more subtle and, in F1 males, exhibits a clear transcriptionally biased regulation.

#### 2.5.2. RNA–Protein Concordance in Enriched GO Terms Associated with Reproductive Processes

Analysis of concordance between transcriptomic and proteomic datasets revealed a differential regulatory pattern of reproductive processes in *S. senegalensis*, with comparison-specific variations depending on sex and origin (Table S2). In the F1F vs. F1M comparison, a balanced set of concordant terms was identified, including both upregulated and downregulated categories, reflecting a clear functional separation between male and female gametogenesis. Among the concordantly upregulated terms, acrosome reaction, binding of sperm to the zona pellucida, and sperm–egg recognition were particularly prominent. These functions are closely associated with fertilization and gamete recognition, suggesting a coordinated transcriptional and proteomic activation of sperm-related mechanisms in F1 males. In contrast, concordantly downregulated terms included oocyte maturation, female gamete generation, and embryonic development, indicating parallel repression of pathways related to oocyte development.

In the F1F vs. wtF comparison, concordance was mainly associated with processes such as gamete generation, spermatid differentiation, and embryonic organ morphogenesis, suggesting a moderate but coordinated omic-level response between F1 and wild-type females. By contrast, the F1M vs. wtM comparison exhibited the most pronounced pattern of concordant repression, characterized by simultaneous downregulation of genes and proteins enriched in GO terms such as fertilization, sexual reproduction, embryonic morphogenesis, and female gamete generation. This pattern indicates a potential impairment of essential reproductive functions in F1 males relative to wild-type counterparts.

Finally, in the wtF vs. wtM comparison, concordantly downregulated terms reflected the typical sexual dimorphism of the species, whereas concordantly upregulated terms, including embryonic development and pattern specification, suggest conserved transcriptional and proteomic control of embryonic differentiation programs.

Overall, the presence of concordant GO terms at both omic levels highlights the existence of integrated regulation of reproductive processes in *S. senegalensis*. However, the coordinated downregulation observed in the F1M vs. wtM comparison suggests a functional alteration that may contribute to the reduced fertility commonly observed in cultured F1 males.

### 2.6. Functional Analysis Based on KEGG Pathways

**Figure 7:**
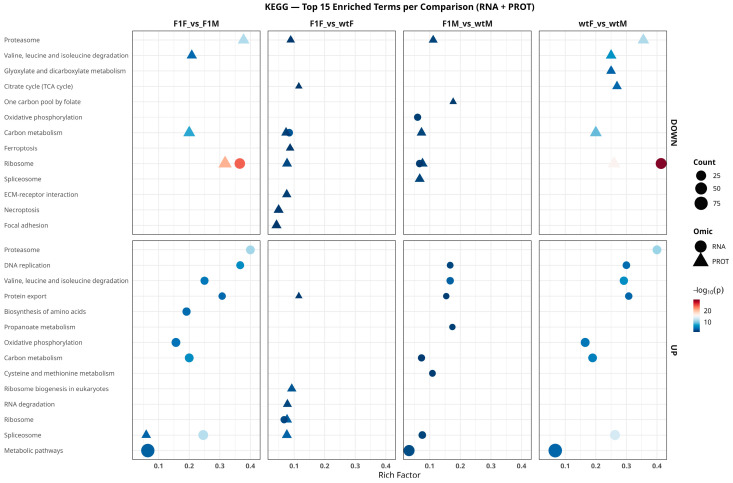
Functional enrichment analysis of KEGG pathways integrating transcriptomic (RNA) and proteomic (PROT) data in *Solea senegalensis*. The most significant KEGG pathways that are positively regulated (UP, **lower panels**) and negatively regulated (DOWN, **upper panels**) in the comparisons F1F vs. F1M, F1F vs. wtF, F1M vs. wtM, and wtF vs. wtM are shown. Circles represent pathways derived from the transcriptome (RNA), whereas triangles correspond to the proteome (PROT). The x-axis indicates the rich factor (proportion of genes/proteins annotated to each pathway relative to the total), symbol size reflects the number of enriched entities (count), and the color scale represents the level of statistical significance (−log_10_ adjusted *p*-value). F1F: first-generation captive-bred female, wtF: wild-type female, F1M: first-generation captive-bred male, and wtM: wild-type male.

Functional enrichment analysis of KEGG pathways (Figure 7) revealed a highly conserved pattern between the transcriptome and proteome, dominated by metabolic and protein synthesis-related pathways, although with clear differences in the direction of regulation and the predominant omic level. Overall, the most significantly shared pathways included Ribosome, Spliceosome, Proteasome, Oxidative phosphorylation, Carbon metabolism, Valine, leucine and isoleucine degradation, and Focal adhesion. These pathways were detected across multiple comparisons, indicating that biosynthetic, degradative, and energetic control mechanisms coexist as central axes in the gonadal physiology of *S. senegalensis*.

### 2.7. DNA Methylation Landscape in Relation to Transcriptomic–Proteomic Patterns

RRBS-based methylation analysis was applied to gonadal samples from wild-type and captive-bred first-generation (F1) females and males to contextualize epigenetic variation within the current transcriptomic–proteomic framework while ensuring consistency with previously reported datasets. RRBS libraries yielded approximately 2.1 × 10^9^ paired-end reads, with high sequencing quality (Q20/Q30 > 85%) and mapping efficiencies of ~62–67%. After quality filtering, methylation was detected in ~53–54% of the analyzed CpG sites, and differential methylation analysis was restricted to CpGs with sufficient coverage (>8 counts), variability, and statistical support after multiple-testing correction (FDR < 0.01). Table S4 and Figure S3 present the global methylation statistics and multidimensional scaling of global methylation profiles.

Methylation profiles segregated primarily by sex, with additional differentiation between F1 and wild-type individuals within each sex, particularly among males. Multidimensional scaling confirmed sex as the main source of epigenetic variation, in agreement with the dominant sex-driven patterns observed at both transcriptomic and proteomic levels.

Origin-dependent structuring was maintained in sex-specific analyses. The highest number of differentially methylated CpGs was observed in contrasts based on rearing origin, followed by sex-based comparisons, while methylation differences remained of similar magnitude across all contrasts (log_2_ fold change ~1.0–1.6).

Differentially methylated CpGs were predominantly located in intronic and intergenic regions, with smaller shifts in promoter-associated sites and a notable enrichment within first introns, indicating that most methylation differences were not concentrated at promoter regions. F1 males showed a higher number of hypermethylated CpGs compared to wild-type males, a trend also observed in females, while sex-based comparisons consistently revealed higher overall methylation levels in females. Overall, the methylation landscape observed here was consistent with previously described patterns in *S. senegalensis* and was therefore used as an epigenetic reference framework rather than subjected to de novo functional annotation.

## 3. Discussion

Our results demonstrate that sex is the biological variable that most clearly structures molecular variation in the gonads of *S. senegalensis*, exerting a stronger effect than origin (F1 vs. wild-type). In the principal component analysis (PCA), the transcriptomic dataset restricted to the matched gene–protein set clearly separated females and males, with the first two components explaining 84.3% of the total variance. A similar sex-based separation was observed in the proteomic dataset, although it explained a lower proportion of the variance (40.1%). This pattern was also reflected in the differential expression analyses, as sex-based comparisons (F1F vs. F1M; wtF vs. wtM) yielded the highest numbers of differentially expressed genes (DEGs) and proteins (DEPs), far exceeding the differences associated with origin. The predominance of sex-driven molecular differences is consistent with multiple studies in fish gonads, where sex has been identified as the main source of transcriptomic and proteomic variation, including in *Oreochromis niloticus* [12] and *Dicentrarchus labrax* [13].

In agreement with previous studies in teleost fishes [14,15], our findings suggest molecular regulation of *S. senegalensis* gonads is strongly influenced by the hypothalamic–pituitary–gonadal (HPG) axis. This endocrine control appears to exert a dominant modulatory effect over genetic or environmental differences between F1 and wild-type individuals, which may explain the functional homogeneity observed in core gonadal processes despite variation in origin.

In contrast to the pronounced sex-related effects, molecular differences associated with origin (F1 vs. wild-type) were comparatively subtle, although biologically informative. In females, the F1F vs. wtF comparison yielded a limited number of DEGs and DEPs, whereas even fewer changes were detected in the F1M vs. wtM contrast. This pattern indicates that, under captive conditions, the gonadal transcriptome and proteome of F1 individuals do not diverge drastically from wild profiles, consistent with observations in flatfish species such as *Paralichthys olivaceus*, where domestication-related differences are reported to be less pronounced in gonads than in somatic tissues [16]. Nevertheless, despite the moderate global differences between origins, the correspondence between transcriptomic and proteomic levels was low, reflecting a pattern widely documented across organisms in which mRNA abundance explains only a limited fraction of protein-level variation. For instance, Ponomarenko et al. [17] reported transcriptome–proteome correlations rarely exceeding 0.3–0.5, constraining the direct prediction of protein abundance from mRNA levels. Similarly, Kuchta et al. [18] observed that even simplified translation models achieved correlation coefficients below 0.2 for many proteins, largely due to the dynamics of translation and protein degradation.

From a biological perspective, this limited integration can be attributed to several key regulatory mechanisms, including translational efficiency (influenced by mRNA structure, ribosome availability, and tRNA pools), protein half-life, and post-transcriptional regulation mediated by microRNAs, alternative splicing, or post-translational modifications [19]. In our study, the F1M vs. wtM contrast, characterized by a low correlation coefficient (r = 0.187) and only 0.08% concordant gene–protein pairs, suggests that F1 males may experience substantial disruptions in translation or protein stability within critical reproductive pathways. This lack of alignment implies a decoupled regulatory state in which transcripts may be present but fail to be efficiently translated or accumulated as functional proteins. From a statistical standpoint, our data further underscore that even in experiments with relatively high sample numbers, strong correlations between transcriptomic and proteomic datasets should not be expected. This is partly attributable to biological noise arising from heterogeneous cell populations within gonadal tissue, individual variability, and differences in detection efficiency between RNA-seq and LC–MS/MS platforms. Indeed, studies in flatfish exposed to environmental stress have reported Pearson correlation coefficients as low as 0.04–0.12 in transcriptome–proteome comparisons [20,21]. Consequently, the limited integration observed here is unlikely to represent a methodological artifact and instead reflects the inherent complexity of gene regulation in mature gonads under captive breeding conditions.

In this context, the integration of previously published DNA methylation data provides an additional regulatory framework for interpreting the transcriptomic–proteomic decoupling observed in the present study, particularly in captive-bred F1 males. Earlier epigenetic analyses of *S. senegalensis* gonadal tissue identified consistent patterns of differential DNA methylation associated with biological origin and reproductive status, affecting genes involved in gonadal development, gametogenesis, and reproductive function, while revealing heterogeneous relationships between CpG methylation levels and transcript abundance [7,10].

These studies reported a complex methylation–expression landscape, including both positive and negative associations as well as cases lacking significant correlation, especially among genes encoding transcription factors and regulatory elements linked to reproductive processes. In the present multi-omics dataset, functional categories related to gamete generation, fertilization, and early embryonic development were recurrently identified through transcriptomic and proteomic enrichment analyses. Notably, several genes contributing to these categories overlapped with those previously annotated to differentially methylated CpGs, although their regulation was not consistently concordant at the RNA and protein levels, particularly in comparisons involving F1 males.

Importantly, methylation changes previously described were predominantly located in intronic and intergenic regions and exhibited moderate effect sizes, suggesting fine-scale regulatory modulation rather than binary transcriptional on/off control. Together, these observations support the view that reproductive gene networks in captive-bred males may remain transcriptionally active while failing to achieve proportional protein-level output. Without implying a direct causal role of DNA methylation, previously described epigenetic patterns offer a plausible regulatory context for the non-linear relationships observed between epigenetic variation, transcript abundance, and protein accumulation in this study. This integrative perspective reinforces the value of multi-omics approaches for dissecting the molecular basis of reproductive dysfunctions associated with captivity in flatfish species.

Despite the limited quantitative correlation between transcriptomic and proteomic datasets, functional integration revealed substantial biological concordance at the level of Gene Ontology (GO) terms, indicating that essential processes maintain coordinated regulation across transcriptional and translational layers in *S. senegalensis*. GO Biological Process analysis showed that the most consistently enriched categories across comparisons were related to translation, ribosome biogenesis, protein assembly and folding, and small-molecule metabolism. The prominence of these biosynthetic and cellular maintenance functions is consistent with the high metabolic and protein synthesis demands of both ovarian and testicular tissues during gamete maturation [22]. In this context, transcriptome–proteome integration revealed particularly strong functional concordance in sex-based comparisons, suggesting that transcriptional regulation is largely reflected at the protein level. Moreover, integrative functional analysis highlighted significant concordance in reproductive processes, supporting coordinated regulation across omic layers.

In the sex-based contrasts (F1F vs. F1M and wtF vs. wtM), concordant genes and proteins were associated with key processes such as gametogenesis and fertilization. By contrast, origin-based comparisons (F1M vs. wtM and F1F vs. wtF) showed coordinated underexpression of reproductive-related terms, pointing to molecular decoupling associated with captive origin In the F1F vs. F1M comparison, concordantly upregulated terms such as acrosome reaction, sperm–egg recognition, and binding of sperm to the zona pellucida reflect the expected sexual dimorphism between female and male gonads. These terms indicate the activation of fertilization-related molecular programs regulated by key proteins, including acrosin and zona pellucida glycoproteins, whose coordinated expression is essential for successful fertilization in teleosts, rather than evidence of altered spermatogenesis [23]. Conversely, concordantly downregulated terms, including oocyte maturation, female gamete generation, and embryonic development, reflect repression of oogenic and embryogenic programs, likely driven by sex-specific hormonal dynamics [24].

Enriched terms were largely linked to gamete generation and germ cell differentiation in the F1F vs. wtF comparison. Although some annotations include spermatid differentiation-related terms due to conserved orthology, they reflect shared molecular programs involved in germ cell maturation rather than male-specific spermatogenic processes in females. These results suggest that F1 females largely maintain the baseline expression of genes involved in gamete maturation, while subtle regulatory differences may influence oocyte quality under captive conditions [25].

The most striking pattern emerged in the F1M vs. wtM comparison, where concordant downregulation of fertilization, sexual reproduction, embryonic development, and gamete generation was observed at both transcriptomic and proteomic levels. This coordinated repression points to a systemic dysfunction of reproductive pathways in F1 males, likely linked to endocrine or/and epigenetic alterations associated with captivity. Previous studies in *S. senegalensis* have shown that F1 males exhibit reduced sperm production and altered seminal fluid proteomic composition, impairing sperm motility and fertilization capacity [4]. In other species, such as *Sparus aurata* and *Dicentrarchus labrax*, coordinated underexpression of genes and proteins related to spermatogenesis has been interpreted as evidence of incomplete gonadal maturation or impaired spermiation [26,27,28].

Furthermore, the concordant downregulation of processes such as embryonic morphogenesis and chordate embryonic development in the F1M vs. wtM comparison suggests that reproductive alterations may extend beyond gametogenesis, potentially affecting early embryonic developmental pathways. This interpretation is supported by observations in flatfish species such as *Scophthalmus maximus* and *Paralichthys olivaceus*, where sperm dysfunction in later-generation captive individuals has been associated with coordinated repression of embryonic development and morphogenesis pathways [27,29,30].

In the wtF vs. wtM comparison, concordant downregulated terms such as oocyte maturation and fertilization reflect expected sexual dimorphism, whereas upregulated terms related to embryonic development and pattern specification indicate the preservation of fundamental developmental programs. Overall, the presence of concordant functional terms across all contrasts supports the notion that reproductive processes in *S. senegalensis* are regulated through an integrated transcriptomic–proteomic framework. However, the coordinated underexpression observed in F1M vs. wtM highlights a profound molecular alteration in the gonads of F1 males, compromising fertilization capacity and potentially explaining the reduced reproductive success observed under captive conditions.

## 4. Materials and Methods

### 4.1. Sampling, Extraction of RNA and Proteins from Gonadal Tissue

Gonadal samples were obtained from four main groups: captive-bred first-generation (F1) females and males (F1F, F1M) and wild-type females and males (wtF, wtM). Wild individuals were collected from the coastal areas of the Bay of Cádiz (Andalusia, southern Spain; approximately 36.10–37° N, 6.35–6.15° W) through authorized trawling activities carried out by local fishermen, who provided the specimens for scientific purposes. The seawater in this location ranges from 14 to 15 °C in winter to 23–24 °C in summer. For transcriptomic analysis (RNA-seq), a total of 19 libraries were generated, including five samples from F1F, five from F1M, five from wtF, and four from wtM. For proteomic analysis (LC–MS/MS), 18 samples were quantified, corresponding to five biological replicates in F1F, five in F1M, five in wtF, and three in wtM (Table 1).

Gonads were excised, and 0.5 mg of tissue was collected for the simultaneous extraction of RNA and proteins using TRIzol^®^ reagent (Invitrogen, Carlsbad, CA, USA), following the manufacturer’s instructions with minor modifications. This approach enabled the parallel recovery of total RNA and protein fractions from the same biological material, ensuring direct comparability between transcriptomic and proteomic datasets. RNA integrity was assessed using a Bioanalyzer 2100 (Agilent Technologies, Santa Clara, CA, USA), and only samples with an RNA Integrity Number (RIN) ≥ 7 were retained for downstream analyses.

### 4.2. RNA Sequencing

RNA-seq libraries were prepared using the TruSeq Stranded mRNA kit (Illumina, Inc., San Diego, CA, USA) and sequenced on an Illumina HiSeq 4000 platform, generating 150 bp paired-end reads. Raw reads were processed with Cutadapt to remove adapter sequences and low-quality reads and subsequently aligned to the *S. senegalensis* reference genome (GCF_019176455.1) using HISAT2. Transcript assembly was performed with StringTie, and gene expression levels were quantified as fragments per kilobase of transcript per million mapped reads (FPKM). Differential gene expression analysis was conducted in R using DESeq2 (v1.40.2), considering genes as differentially expressed (DEGs) when |log_2_ fold change (LFC)| ≥ 1 and false discovery rate (FDR) < 0.05.

### 4.3. Protein Analysis by LC-MS/MS

In parallel, total proteins were quantified using the bicinchoninic acid (BCA) assay. Protein quality was evaluated by SDS–PAGE using 10% acrylamide gels, followed by Coomassie Blue staining. Samples were subjected to reduction with 20 mM dithiothreitol (DTT), alkylation with 40 mM iodoacetamide (IAA), and enzymatic digestion with trypsin. The resulting peptides were desalted using C18 columns and analyzed on an Orbitrap Fusion LC–MS/MS system (Thermo Fisher Scientific, Waltham, MA, USA).

Raw mass spectrometry files were processed using PEAKS Studio Xpro and MaxQuant (v2.0.3.1), employing the *S. senegalensis* RefSeq protein database. Protein quantification was based on label-free quantification (LFQ) intensities, and differential protein expression analysis was performed in R using the limma package (v3.58.1). Proteins were considered differentially expressed (DEPs) when log_2_FC ≥ 0.5 and FDR < 0.05.

### 4.4. Bioinformatic Analysis for Omics Integration and Functional Enrichment

Transcriptomic–proteomic integration was performed in R (v4.3.1). The 2221 identified proteins were mapped to their corresponding genes using a custom gene–protein correspondence file (Table S3), thereby restricting downstream analyses to the matched gene–protein set. Within this dataset, DEGs and DEPs were intersected to identify concordant and discordant expression patterns, enabling the evaluation of consistency between mRNA and protein levels.

To incorporate an epigenetic regulatory layer into the present multi-omics framework, DNA methylation analysis was performed following the same experimental design and analytical workflow previously described for gonadal tissue of *S. senegalensis* [7]. Methylation profiling was conducted on gonadal tissue from the same four experimental groups analyzed at the transcriptomic and proteomic levels: captive-bred first-generation females and males (F1F, F1M) and wild-type females and males (wtF, wtM).

Genome-wide DNA methylation profiles were obtained by reduced representation bisulfite sequencing (RRBS). Briefly, raw sequencing reads were assessed using FastQC, adapter- and quality-trimmed with Trim Galore, and aligned to the *S. senegalensis* reference genome (GCF_019176455.1) using Bismark. CpG-level methylation calls were extracted using the same parameters, filtering thresholds, and analytical criteria applied in the reference study. The resulting methylation profiles were examined at the global level to characterize the epigenetic landscape of the individuals included in the present study and to support the functional interpretation of transcriptomic and proteomic patterns using previously reported methylation-associated gene annotations.

Functional enrichment analysis was performed in R (v4.3.2) using the gprofiler2 package (v0.2.2) to retrieve Gene Ontology (GO) terms (Biological Process, Molecular Function, and Cellular Component) and KEGG pathways. This rank-based approach, using gene and protein lists ordered by log_2_FC, allowed the detection of coordinated enrichment patterns across functional categories, even in comparisons with a limited number of differentially expressed entities, thereby providing a more robust and sensitive view of regulated pathways. Results were visualized using ggplot2 (v3.4.4), generating bubble plots and comparative panels to distinguish upregulated (UP) and downregulated (DOWN) categories.

## 5. Conclusions

The integration of transcriptomic and proteomic data in gonads of *S. senegalensis* revealed a partial correspondence between mRNA and protein levels, indicating that gene regulation in this tissue is governed by complex control mechanisms that extend beyond transcription. Despite the moderate correlations observed, functional analyses demonstrated substantial biological coherence, particularly in pathways related to gametogenesis, fertilization, and early embryonic development. These findings reflect a coordinated regulation of reproductive processes across multiple layers of molecular control.

Comparisons between sexes showed relatively high functional concordance, whereas contrasts based on origin (F1 vs. wild type) exhibited a more pronounced decoupling, associated with the coordinated under-expression of reproductive-related terms in F1 males. This pattern suggests that impairments in maturation and reproductive efficiency observed in cultured individuals are more likely driven by post-transcriptional and post-translational regulatory mechanisms rather than by differences in gene transcription alone. Additionally, the integration of previously characterized DNA methylation patterns provides an additional regulatory context for these observations, supporting the view that epigenetic modulation may contribute to the observed transcriptome–proteome decoupling without necessarily disrupting baseline transcriptional profiles.

Overall, the results of this study highlight the value of a multi-omics approach for elucidating the regulation of gonadal function and the molecular basis of reproductive dysfunction in aquaculture species. Furthermore, they provide a robust foundation for the development of strategies aimed at improving maturation, fertility, and reproductive performance in captive-bred F1 populations.

## Figures and Tables

**Table 1:** Number of transcriptomic (RNA-seq) and proteomic (LC–MS/MS) samples analyzed in gonads of *Solea senegalensis*. Female (F) and male (M) individuals from both captive-bred first-generation (F1) and wild-type (wt) groups are included.

Samples	RNA-Seq	Proteomics	Groups
F1F1, F1F2, F1F3, F1F4, F1F5	5	5	F1F
F1M1, F1M2, F1M3, F1M4, F1M5	5	5	F1M
wtF1, wtF2, wtF3, wtF4, wtF5	5	5	wtF
wtM1, wtM2, wtM3, wtM4	4	3	wtM

## Data Availability

The mass spectrometry proteomics data have been deposited to the ProteomeXchange Consortium via the PRIDE (1) partner repository with the dataset identifier PXD071796.

## Supplementary Materials

The following supporting information can be downloaded at: https://www.mdpi.com/article/10.3390/ijms27052153/s1.

## Abbreviations

The following abbreviations were used in this manuscript:

BP	Biological Process
BCA	Bicinchoninic Acid
CC	Cellular Component
CpG	Cytosine–phosphate–guanine dinucleotide
DEG	Differentially Expressed Gene
DEP	Differentially Expressed Protein
DTT	Dithiothreitol
F1	First-generation captive-bred
F1F	First-generation captive-bred Female
F1M	First-generation captive-bred Male
FDR	False Discovery Rate
FPKM	Fragments per Kilobase of transcript per Million mapped
GO	Gene Ontology
IAA	Iodoacetamide
KEGG	Kyoto Encyclopedia of Genes and Genomes
LC–MS/MS	Liquid Chromatography–Tandem Mass Spectrometry
LFQ	Label-Free Quantification
MF	Molecular Function
NES	Normalized Enrichment Score
PCA	Principal Component Analysis
RNA-seq	RNA sequencing
RRBS	Reduced Representation Bisulfite Sequencing
SDS-PAGE	Sodium Dodecyl Sulfate–Polyacrylamide Gel Electrophoresis
UP/DOWN	Overexpressed/Underexpressed
Wt	Wild-type
wtF	Wild-type Female
WTM	Wild-type Male
