# Supplementary material for: Integrative Transcriptomic, Proteomic and Epigenetic Analysis Uncovers Reproductive Dysregulation in F1 Males of Solea senegalensis
Source: Int J Mol Sci. 2026 Feb 25;27(5):2153. doi: 10.3390/ijms27052153 (PMC12984382; doi:10.3390/ijms27052153)
Supplement: Supplementary file 1 [file ijms-27-02153-s001.zip › Figure S3.pptx]

## Slide 1
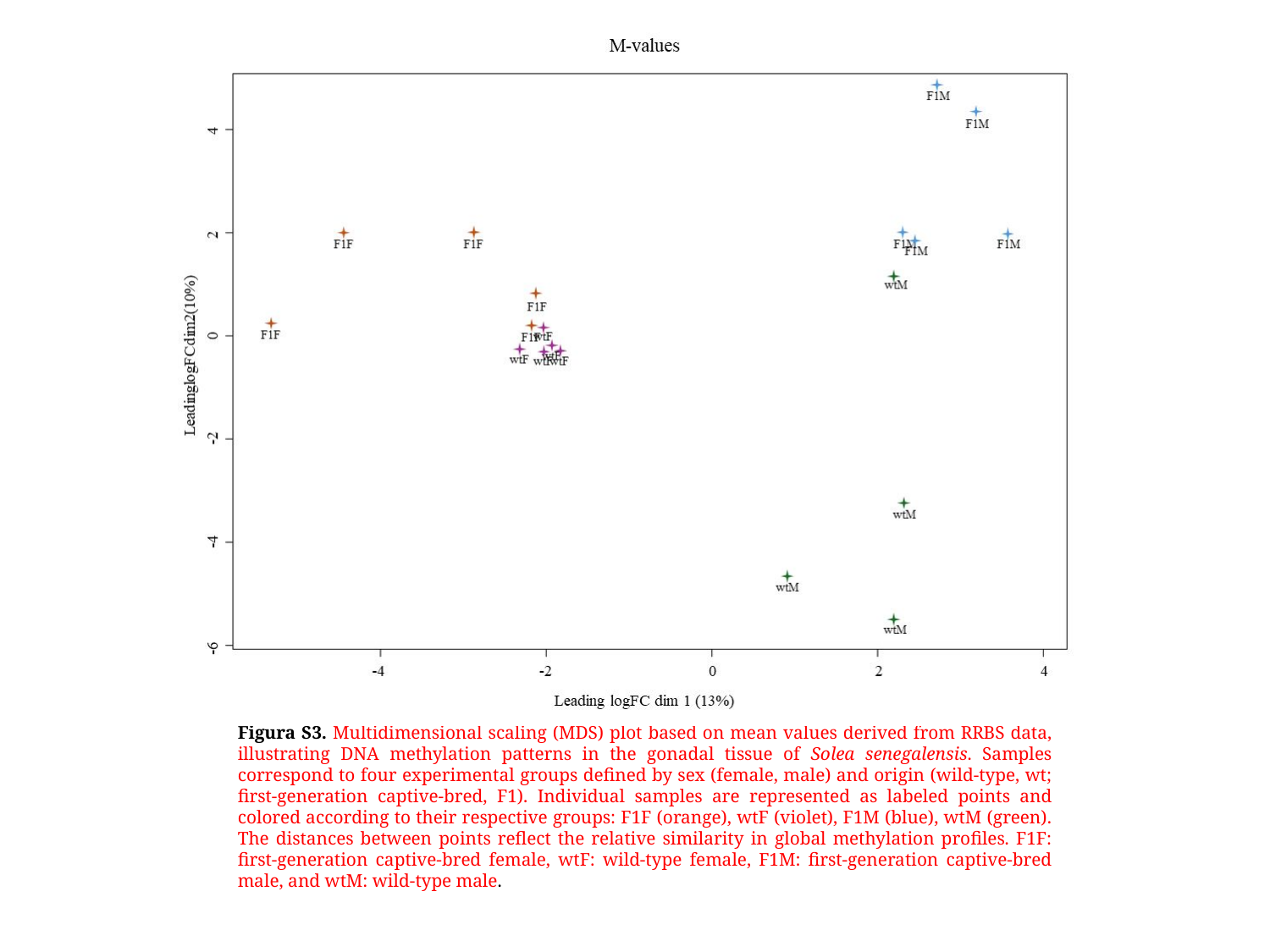

Figura S3. Multidimensional scaling (MDS) plot based on mean values derived from RRBS data, illustrating DNA methylation patterns in the gonadal tissue of Solea senegalensis. Samples correspond to four experimental groups defined by sex (female, male) and origin (wild-type, wt; first-generation captive-bred, F1). Individual samples are represented as labeled points and colored according to their respective groups: F1F (orange), wtF (violet), F1M (blue), wtM (green). The distances between points reflect the relative similarity in global methylation profiles. F1F: first-generation captive-bred female, wtF: wild-type female, F1M: first-generation captive-bred male, and wtM: wild-type male.
