# Supplementary material for: Integrative Transcriptomic, Proteomic and Epigenetic Analysis Uncovers Reproductive Dysregulation in F1 Males of Solea senegalensis
Source: Int J Mol Sci. 2026 Feb 25;27(5):2153. doi: 10.3390/ijms27052153 (PMC12984382; doi:10.3390/ijms27052153)
Supplement: Supplementary file 1 [file ijms-27-02153-s001.zip › Supplementary Materials Legends-vAAP.pdf]

Supplementary Materials Legends

Table S1. Table S1. General RNA-seq sequencing statistics of the gonadal transcriptome of wild-type (wt) and first-generation captive-bred (F1) *Solea senegalensis*, including females (F) and males (M). The table summarizes the total number of raw reads (raw_reads), raw bases (raw_bases), clean reads after quality filtering (clean_reads), clean bases (clean_bases), sequencing error rate (error_rate), percentage of bases with quality scores ≥ Q20 and ≥ Q30, and GC content (GC_pct). F1F: first-generation captive-bred female, wtF: wild-type female, F1M: first-generation captive-bred male, and wtM: wild-type male; 1 - 5 are the number of the samples analyzed in each group.

Table S2. The table summarizes Gene Ontology (GO) biological process terms related to reproduction, gametogenesis, fertilization, and embryonic development identified through integrated transcriptomic–proteomic analysis of *Solea senegalensis*. The comparisons include F1F vs. F1M, F1F vs. wtF, F1M vs. wtM, and wtF vs. wtM. For each GO term, the direction of regulation at the RNA and protein levels is indicated, together with its classification as concordant or discordant between both omic layers. F1F: first-generation captive-bred female, wtF: wild-type female, F1M: first-generation captive-bred male, and wtM: wild-type male.

Table S3. This table presents the customized correspondence file linking protein identifiers (RefSeq XP accessions) obtained by LC–MS/MS to their associated gene identifiers (gene_id). In cases where multiple protein isoforms correspond to a single gene, they were collapsed into a unique gene-level entry. This mapping was used to integrate transcriptomic and proteomic datasets, enabling joint multi-omics analyses based on a shared gene universe.

Table S4. Summary of cytosine methylation calls obtained from RRBS analysis. Values are presented as means across the samples. Total counts were first calculated per sample and subsequently averaged for each category. Cytosines were classified based on their sequence context (CpG, CHG, CHH, or unknown). The percentages indicate the proportion of methylated cytosines relative to the total number of cytosines analyzed within each context. F1F: first-generation captive-bred female, wtF: wild-type female, F1M: first-generation captive-bred male, and wtM: wild-type male.
*For each category, the total counts were first calculated per sample and subsequently averaged.

Figure S1. Panel of enriched GO terms from the Cellular Component category (GO:CC) integrating transcriptomic (RNA-seq) and proteomic (LC–MS/MS) data from gonadal tissues of *Solea senegalensis*. The top 15 enriched terms are shown for each biological comparison and condition (UP and DOWN). The x-axis represents the Rich Factor, point size indicates the number of enriched elements, and color denotes statistical significance ($-\log_{10}$ p). Circles and triangles correspond to transcriptomic and proteomic results, respectively.

Figure S2. Panel of enriched GO terms from the Molecular Function category (GO:MF) integrating transcriptomic (RNA-seq) and proteomic (LC–MS/MS) data from gonadal tissues of *Solea senegalensis*. The top 15 enriched terms are shown for each biological comparison and condition (UP and DOWN). The x-axis represents the Rich Factor, point size indicates the number of enriched elements, and color denotes statistical significance ($-\log_{10}$ p). Circles and triangles correspond to transcriptomic and proteomic results, respectively.

Figure S3. Multidimensional scaling (MDS) plot based on mean values derived from RRBS data, illustrating DNA methylation patterns in the gonadal tissue of *Solea senegalensis*. Samples correspond to four experimental groups defined by sex (female, male) and origin (wild-type, wt; first-generation captive-bred, F1). Individual samples are represented as labeled points and colored according to their respective groups: F1F (orange), wtF (violet), F1M (blue), wtM (green). The distances between points reflect the relative similarity in global methylation profiles. F1F: first-generation captive-bred female, wtF: wild-type female, F1M: first-generation captive-bred male, and wtM: wild-type male.
