# Supplementary figures and images for: Integrative Transcriptomic, Proteomic and Epigenetic Analysis Uncovers Reproductive Dysregulation in F1 Males of Solea senegalensis
Source: Int J Mol Sci. 2026 Feb 25;27(5):2153. doi: 10.3390/ijms27052153 (PMC12984382; doi:10.3390/ijms27052153)

GO:CC ... Top 15 Enriched Terms per Comparison (RNA + PROT)

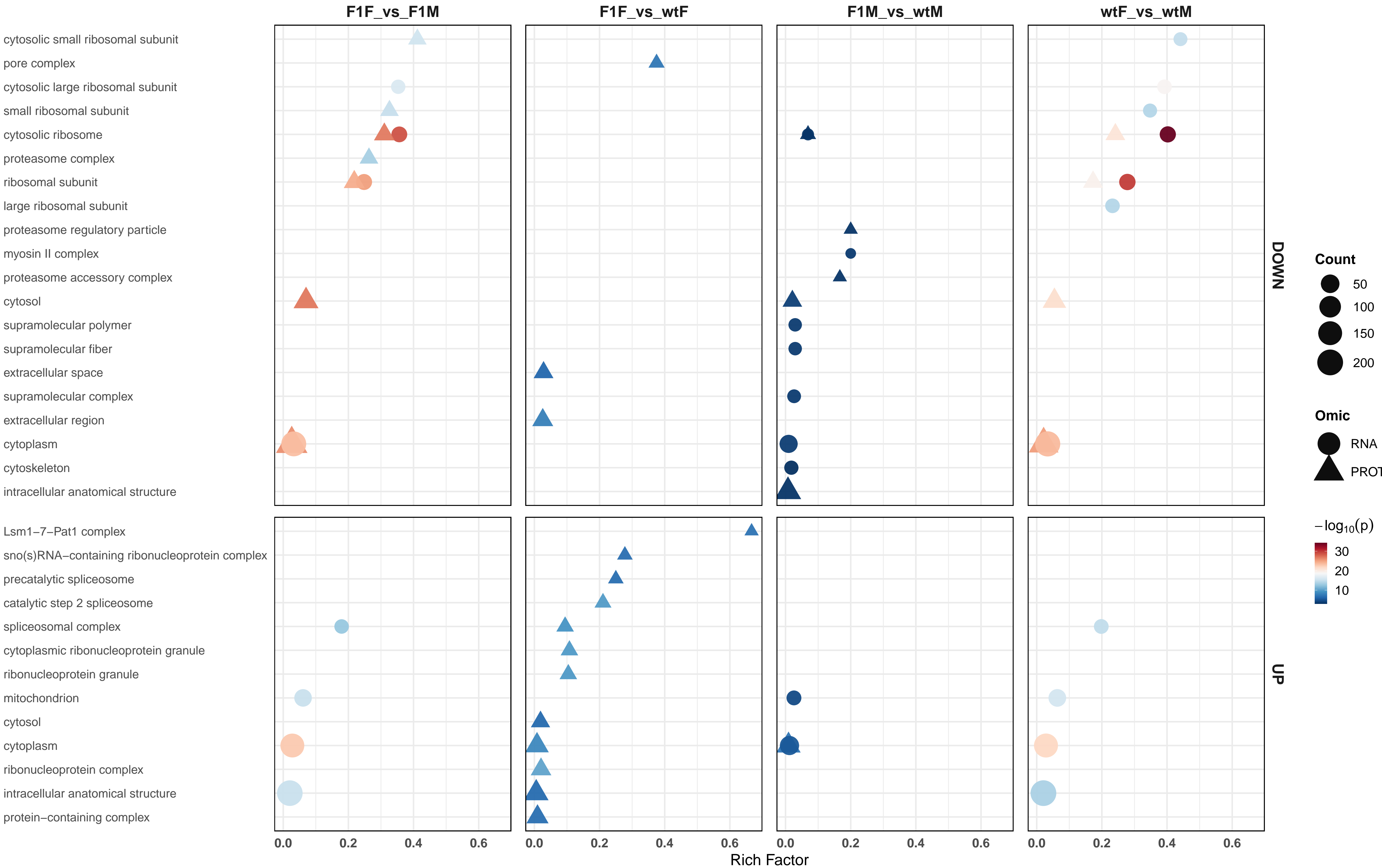

Supplement: Supplementary file 1 [file ijms-27-02153-s001.zip › Figure S1.pdf]

GO:MF ... Top 15 Enriched Terms per Comparison (RNA + PROT)

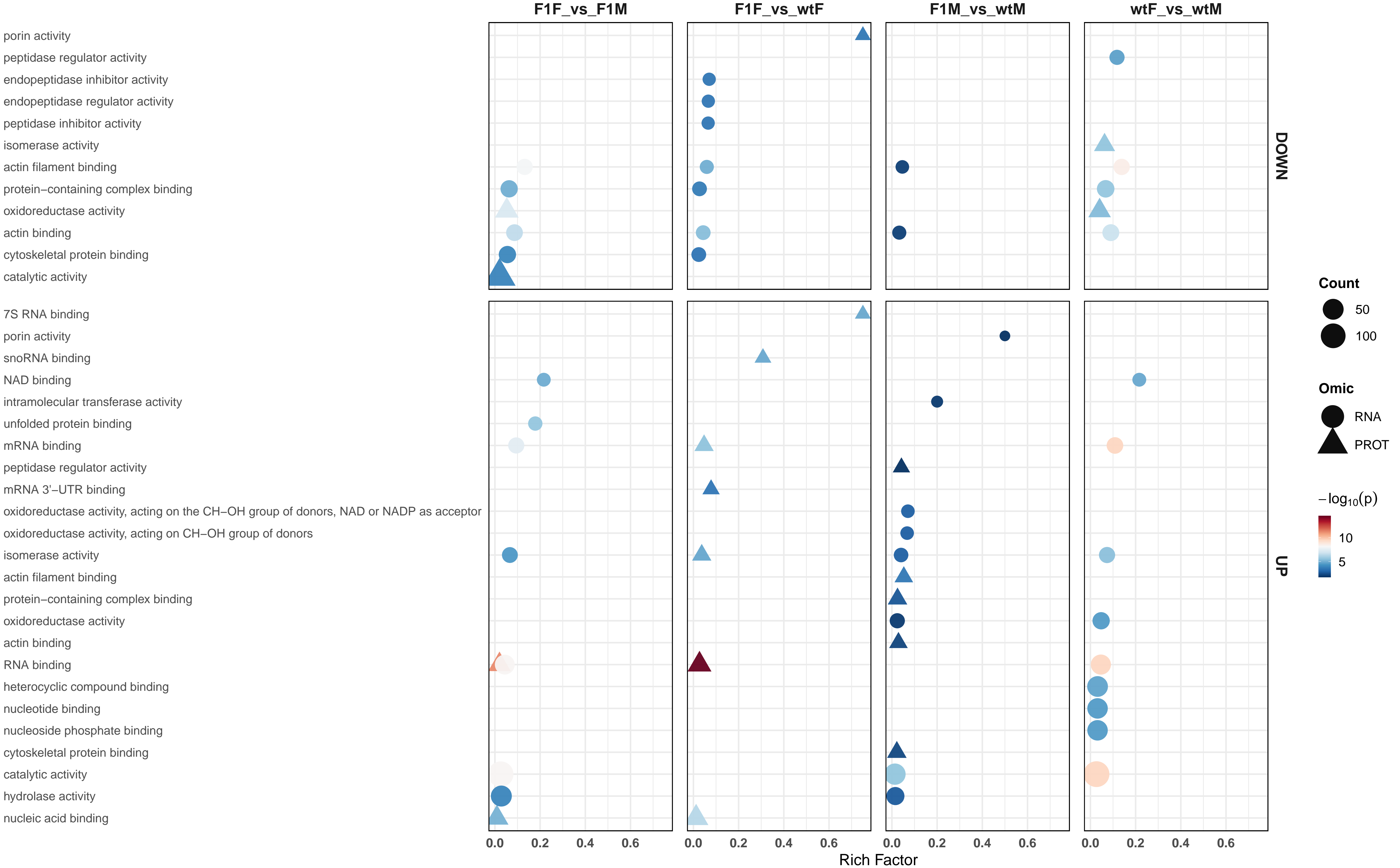

Supplement: Supplementary file 1 [file ijms-27-02153-s001.zip › Figure S2.pdf]
